# New Medical Journal

**Published:** 1868-07

**Authors:** 


NEW MEDICAL JOURNAL.—As we go to press, we have received the first number of the Monthly Medical Reprint, published in New York, by John Hillyer, 14 South William street. This journal proposes to be a reproduction of the most valuable articles published in the latest issues of the British medical journals, and some translations from the French and German medical press. It is to be published monthly at $5 per annum. We have no space to notice the contents of this number, but shall speak of it hereafter.

